# To evaluate if increased supervision and support of South African Government health workers’ home visits improves maternal and child outcomes: study protocol for a randomized control trial

**DOI:** 10.1186/s13063-017-2074-5

**Published:** 2017-08-07

**Authors:** Mary Jane Rotheram-Borus, Karl Le Roux, Ingrid M. Le Roux, Joan Christodoulou, Christina Laurenzi, Nokwanele Mbewu, Mark Tomlinson

**Affiliations:** 1 Department of Psychiatry & Biobehavioral Sciences, Semel Institute, University of California, 10920 Wilshire Blvd., Suite 350, Los Angeles, CA 90024 USA; 2 Zithulele Hospital, P Bag X504, Mqanduli, 5080 South Africa; 3 Philani Maternal, Child Health and Nutrition Project, Elonwabeni, PO Box 40188, 7791 Cape Town, South Africa; 4 Department of Psychology, Stellenbosch University, Private Bag X1, Matieland, 7602 South Africa

**Keywords:** Pregnancy, HIV, Maternal health, Perinatal health, Infants, Mothers, South Africa

## Abstract

**Background:**

Concurrent epidemics of HIV, depression, alcohol abuse, and partner violence threaten maternal and child health (MCH) in South Africa. Although home visiting has been repeatedly demonstrated efficacious in research evaluations, efficacy disappears when programs are scaled broadly. In this cluster randomized controlled trial (RCT), we examine whether the benefits of ongoing accountability and supervision within an existing government funded and implemented community health workers (CHW) home visiting program ensure the effectiveness of home visiting.

**Methods/Design:**

In the deeply rural, Eastern Cape of South Africa, CHW will be hired by the government and will be initially trained by the Philani Programme to conduct home visits with all pregnant mothers and their children until the children are 2 years old. Eight clinics will be randomized to receive either (1) the Accountable Care Condition in which additional monitoring and accountability systems that Philani routinely uses are implemented (4 clinics, 16 CHW, 450 households); or (2) a Standard Care Condition of initial Philani training, but with supervision and monitoring being delivered by local government structures and systems (4 clinics, 21 CHW, 450 households). In the Accountable Care Condition areas, the CHW’s mobile phone reports, which are time-location stamped, will be monitored and data-informed supervision will be provided, as well as monitoring growth, medical adherence, mental health, and alcohol use outcomes. Interviewers will independently assess outcomes at pregnancy at 3, 6, 15, and 24 months post-birth. The primary outcome will be a composite score of documenting maternal HIV/TB testing, linkage to care, treatment adherence and retention, as well as child physical growth, cognitive functioning, and child behavior and developmental milestones.

**Discussion:**

The proposed cluster RCT will evaluate whether routinely implementing supervision and accountability procedures and monitoring CHWs’ over time will improve MCH outcomes over the first 2 years of life.

**Trial Registration:**

ClinicalTrials.gov registration #NCT02957799, registered on October 26, 2016.

**Electronic supplementary material:**

The online version of this article (doi:10.1186/s13063-017-2074-5) contains supplementary material, which is available to authorized users.

## Background

Home visiting has been repeatedly demonstrated efficacious in improving maternal and child health (MCH) outcomes when delivered by nurses [1–3] and paraprofessionals in low- and middle-income countries (LMIC) [4–6]. Each dollar invested in maintaining MCH yields a nine-fold benefit [7, 8]. Yet, when home visiting is scaled by community health workers (CHW), the efficacy often disappears [9]. The Alma Alta Declaration has encouraged attempts to mount CHW interventions in all LMICs, and therefore the strategies needed to consistently implement high quality programs with efficacious outcomes are increasingly important. The proposed study aims to identify how supervision and accountability strategies improve MCH outcomes of home visits by CHW.

In South Africa, the challenges to secure positive child outcomes for MCH remain overwhelming. Between 26% and 30% of mothers are living with HIV (MLH) and, while only a small percentage of their children are going to be seropositive, the uninfected children of MLH have growth and immune function challenges. Approximately 25% of pregnant mothers drink alcohol while pregnant, yielding the highest rates of fetal alcohol syndrome disorder in the world (9.5%) [10–12]. Maternal depression is self-reported by approximately 15% of mothers and 6% report depressive symptoms likely to be clinically significant. In every country, maternal depression impacts on child growth and development lifelong [13–16]. Finally, approximately 16% of mothers experience intimate partner violence, which remains very stable over time [17–22]. These maternal challenges threaten the health of their children. For example, in some South African communities, approximately 17% of babies are born with low birth weights (<2500 g) and these infants will be at lifelong risk for multiple negative outcomes [23–25]. Clearly, the number and scope of the challenges mothers experience indicate the importance of broad, structural implementation of efficacious interventions to support pregnant mothers and that this support needs to be extended over the first 5 years of life.

While paraprofessional home visiting has been repeatedly demonstrated to be efficacious, it has not been scaled nor does it concurrently retain efficacy. African countries have the lowest per capita health budgets and fewest well-trained personnel globally [26–30]. Building the personnel capacity, especially by systematically implementing training and accountability structures, has rarely been addressed [28]. This protocol is targeted at filling this gap.

The Philani Intervention Model (PIM) is a non-government organization with a 30-year history of successful, non-stigmatizing, and sustainable home-based support for pregnant women and malnourished children [17, 21, 31–35]. Key components of the PIM are to recruit CHWs who are community role models, train the CHWs extensively, with ongoing in-service trainings, and to monitor the quality of all contacts with supervisors randomly dropping-in on each CHW’s home visits biweekly and reviewing charts weekly, using the charts to iteratively build on CHWs’ skills. Our planned trial will build on this model, seeing if existing government CHWs (not selected to be role models), can improve the quality of their skills and home visits with a significant impact on MCH outcomes.

Therefore, in this study, the PIM field coordinators and supervisors will assume supervision and support responsibilities for government-hired CHWs in a deeply rural setting in the Eastern Cape of South Africa. CHWs in the PIM are equipped with mobile phones to allow easy accountability; that is, a phone that has a time and location stamp on which the CHW provides ratings of the visit’s content, the skills utilized, child weight/height, and perceived impact. The standard supervision procedures used by PIM will be implemented with CHWs randomized to the PIM condition. Based on the Quality Implementation Model [36], MCH outcomes will be monitored over 24 months to examine if accountability improves CHW outcomes.

## Methods/Design

**Fig. 1:**
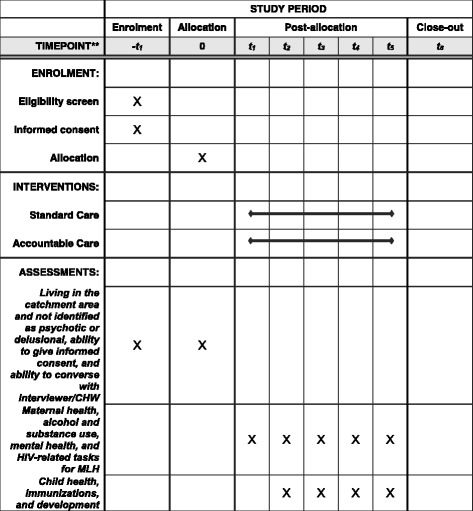
Schedule of enrollment, interventions, and assessments

The Institutional Review Boards of the University of California, Los Angeles (UCLA; IRB#16-001362) and Stellenbosch University (#N16/05/064) have approved the study protocol. A Data Safety Monitoring Board will review interim analyses of the 6- and 15-month assessments prior to the collection of the primary outcome data at 24 months. Each adverse event is reported to both Stellenbosch and UCLA Institutional Review Boards and the Data Safety Monitoring Board. The study protocol was developed in accordance with the Standard Protocol Items: Recommendations for Interventional Trials (SPIRIT) and the populated checklist and figure have been provided (Additional file 1 and Fig. 1). Authorship will be based on standards outlined by the American Psychological Association [37].

### Setting

The OR Tambo District is a rural, hilly area in the Eastern Cape of South Africa accessible on a 150 km paved road east from Umtata and north of East London, with very high unemployment. The area is ancient tribal land, with the tribe allowing only minimal industrialization. Zithulele Hospital serves 130,000 families with nine local clinics. To reach clinics, a patient must cover large distances of hilly terrain either by walking or using the expensive and infrequent mini bus taxis. Aerial maps provided information on the density of each potential study area.

**Fig. 2:**
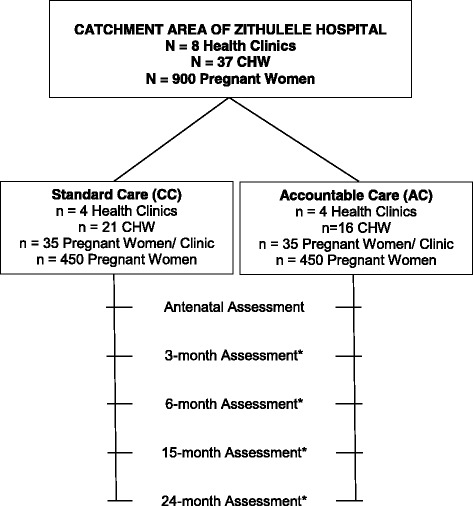
Description of the RCT for area randomization by UCLA to one of two conditions

With the support of the District Health Officer, the team has documented the catchment areas of each CHW assigned to eight clinics in the catchment area of Zithulele Hospital. Data on access to electricity (8% in the region), water tanks (16%), flush toilets (few), distance from the road and the local clinics, and hilliness was used to identify areas of about 200 households each. The areas were matched by UCLA and all CHWs at each clinic (3–5 per clinic) were randomized to either (1) the Accountable Care (AC) condition, in which the PIM supervision and training models will be implemented (4 clinics, 16 CHW, 450 households); or (2) Standard Care (SC) by existing government supervisors and CHWs implementing home visits (4 clinics, 21 CHW, 450 households). Risk of contamination is very low given it is a deeply rural region with vast distances between villages and even homes. Figure 2 outlines the design of the randomized controlled trial (RCT), with the planned follow-up assessments by an independent team of assessors.

### Recruitment

Female research interviewers have been placed in each clinic to recruit all pregnant women and securing voluntary informed consent. Each recruiter covers one clinic in the AC and one clinic in the SC condition. The eligibility criteria to participate in the study will be living in the catchment area and not identified as psychotic or delusional based on the interviewer’s judgment. Exclusion criteria are inability to give informed consent, inability to converse with the interviewer or the CHW, and death of the mother or infant. After consent, an independent team of assessors will conduct the follow-up assessments at the mother’s home.

### Assessments

Independent assessments will be conducted by Stellenbosch University during pregnancy, within 3 months of birth, and at 6, 15, and 24 months post-birth. The PIM is a generalist model. Therefore, there is a cluster of both maternal outcomes and child outcomes. Table 1 summarizes these measures, with the source of the data to determine accomplishment (1) or not (0) of each outcome. Assessors will conduct interviews and gather additional information from health case files, as well as the child’s government-issued, Road-to-Health card. All data will be entered on mobile phones with preprogrammed responses and sent encrypted to a data center. Data for identification will be stored separately from the assessment data. Assessment data will be coded with personal identification numbers and will not include any personally identifiable data. De-identified assessment data will be available through a password-protected, cloud-based database and accessed by UCLA and Stellenbosch University for quality control and, later, analysis. The risk of gathering information with properly trained and supervised staff poses minimal risk to the proposed participants. There are no physical risks associated with participation.

**Table 1:** Outcomes assessed independently during pregnancy, within 3 months of birth, and at 6, 15, and 24 months post-birth scored by the attainment (1) or not (0) of well-being from 10 indicators of maternal and child health

Outcome	Measure
For Mother	
Adherence to medical regimes	Clinic records and self-reports; for mothers living with HIV, there are six prevention of mother-to-child transmission tasks, re-engagement in HIV care post-birth, receipt and adherence to antiretroviral medications over the last week
Breastfed for 6 months (mixed ok)	Self-report and home observations
Alcohol use (AUDIT-C > 2)	Breathalyzer and self-reports
Mental health (EPDS > 13)	Self-reports
For Children	
Growth in height (< −2 SD)	Measurements; tape measures and measuring boards calibrated biweekly
Growth in weight (< −2 SD)	Measurements; scales calibrated biweekly
Adherence to medical regimes	Clinic records, self-report, including for diarrhea, immunizations, respiratory illnesses
Number of hospitalizations and clinic visits	Clinic records, self-reports
WHO Developmental scale in normal range	Assessment of child and maternal reports
Bayley scale in normal range (24 months only)	Assessment of the child
Child Behavior Checklist in normal range on all scales	Maternal reports

Alcohol Use Disorders Identification Test-Clinical Utility (AUDIT-C)

Edinburgh Perinatal Depression Inventory (EPDS)

### Primary outcomes

Self-reports, the Road-to-Health card, and real-time reports by CHWs on mobile phones and in folders will be sources of data. Data collected from the assessment team will always be the gold standard, compared to data from the CHWs. Observational data will be seen as more valid and reliable than maternal self-report data. In the detailed analyses of each outcome with more than one data source, a sensitivity analysis will be conducted to examine whether a different result would have been observed if a different informant had been used as the outcome measure. The existing CHW reporting system for the government is a log of visits, with little more information than indicating that a visit occurred. The implementation design will not allow this RCT to improve this system and a key aspect of this study is whether the monitoring and data-informed supervision make a significant difference on MCH outcomes.

### Maternal assessments

Well-baby clinics provide mothers with a Road-to-Health card that is taken to each healthcare visit and includes information about immunizations and basic health data. All mothers are entitled to ZAR300 monthly as a child grant and the majority of mothers obtain this grant by 24 months after birth in the Eastern Cape [38, 39].

#### General health

Maternal adherence to medical regimens in pregnancy will be monitored, as well as HIV testing in pregnancy, receiving the results at a clinic visit at 1 week post birth, and prenatal vitamins. Adherence of the mother to prescribed healthcare and health routines for herself, her baby, and her partners will be assessed. Alcohol and substance use will be monitored over time, including any alcohol use in pregnancy, frequency of use, the number of drinks per use, and withdrawal symptoms using the three-item AUDIT-C [40, 41], a standardized measure with scores > 2 indicating problematic drinking; scored as 1 if absent, 0 if present.

#### Mental health

The Edinburgh Perinatal Depression Inventory [42], a 10-item scale, will yield the primary depression score and classification as depressed if the score is over 13. Additional maternal health measures are included, but since they are not part of the primary outcome, are not listed here.

#### HIV-related tasks for MLH

MLH have six tasks to accomplish during pregnancy and additional tasks for the first 6 months post-birth related to their children, which will be rated 0 (non-occurring) or 1 (occurring) for each task. These tasks are to take highly active antiretroviral therapy (ARV) from the moment they are diagnosed with HIV irrespective of CD4 count (and continuing for life as per the SA guidelines); have at least one viral load (VL) tested during pregnancy which is less than 400 copies/ mL and have VL taken at least monthly until the cessation of breastfeeding; give child Nevirapine syrup for 6 weeks post-birth (or Nevirapine and AZT syrup for 12 weeks if deemed high risk by a clinician); get child HIV tested with PCR at birth and at 10 -14 weeks post delivery; and, breastfeed exclusively for 6 months. MLH will also report to who, when, and how their HIV status was disclosed. ARV adherence, and their child’s ARV adherence until receipt of their HIV test results, method of feeding, and whether only breastfeeding for 6 months occured will also be recorded. Information on MLHs’ strategy to protect their partner will be collected. MLH must continue HIV care post delivery at their hospital or local clinic following childbirth, have their VL, creatinine, and CD4 monitored, and take ARV consistently.

### Child measures

#### General health

Children’s physical growth is reflected in their age-standardized weight, height, weight-for-height, and head circumference based on the World Health Organization’s (WHO) age-adjusted norms (http://www.who.int/childgrowth/standards/en/) [43]. We will use the child’s government-issued Road-to-Health card and hospitalizations at each assessment point. Well-baby clinic visits should include a critical visit at the 1-week post-birth follow-up.

#### Immunizations

Recommended visits on baby’s health card with immunizations for diphtheria, tetanus, pertusis, HIV, polio, measles, and hepatitis B prior to 24 months will be recorded.

#### Development

The WHO developmental milestones for children at 12 (four tasks) and 24 months (six tasks) and the motor and speech developmental milestones will be administered at 12 months (four tasks) and 24 months (six tasks) [44]. The Bayley Scales of Infant and Toddler Development, Third Edition will be used to assess the developmental outcome of the children at 24 months corrected for age (for prematurity) [45]. The child is tested on three major aspects of development, namely cognition, language, and motor development. The scale has good test-retest reliability and concurrent validity when compared to other infant tests among South African children [46] and with HIV-positive babies on ARV. At 24 months, we will also monitor the children’s behavioral and emotional problems using the Child Behavior Checklist [47–49]. Additional child measures are included, but since they are not part of the primary outcome, are not listed here.

### Intervention

#### SC

All CHWs will receive a mobile phone and initial Philani training for conducting home visits. The CHW will visit the mothers twice monthly during pregnancy until children are 6 months and then monthly until the children reach 2 years of age. All mothers are asked to re-enroll annually with voluntary informed consent at each assessment. The initial messages to CHWs with pregnancy and new mothers and the first 6 months of life are (1) HIV testing, adherence to therapy for MLH, nevirapine at childbirth, hospital delivery, a single feeding method for 6 months, PCR infant HIV testing at 6 weeks, and MLH reengagement in care and adherence to medication; (2) breastfeeding solely for 6 months, monitored by charting growth at each visit, and problem solving on how and where to get food; (3) reducing and eliminating alcohol use; (4) getting the child grant; (5) engaging in pleasant activities and building a social network; and (6) attending clinic visits (four antenatal visits, Well-Child visits, immunizations). After the first 6 months, the CHW focuses on encouraging mothers to stimulate and support their children daily.

Households in the target areas will be visited by government-funded CHWs. This is currently the SC in the Eastern Cape in the wards adjacent to the Zithulele catchment area (although not in all of the Eastern Cape). The existing government-implemented training, monitoring, and supervision structures will remain in place with the only addition being an initial Philani training for conducting home visits. The geographic areas covered by each of the existing CHWs in two wards will be defined and all pregnant women coming to the clinics will be recruited with voluntary informed consent by paraprofessionals or the assessment team. CHWs in the SC will refer all mothers to local clinics for HIV, blood sugar, and pregnancy testing. The rates of visits and tests are available from clinic data and also will be individually collected by the assessment team.

#### AC

The CHWs in the AC will receive the initial Philani training as described above. In addition, the CHWs’ implementation will be consistently monitored and an accountability system will be established. In AC, supervisors will randomly observe home visits once every 2 weeks. CHWs in the AC will log their home visits on their mobile phones, including a rating of the content and skills addressed on the visit, children’s height and weight, and report on achievement of outcomes such as receiving the child grant, immunizations, breastfeeding, and retention and adherence to HIV care. The supervisors have two types of information, (1) personal observations of the CHW as they accompany the CHWs on their visits for 1 day every 2 weeks; and (2) reviewing data from a console as well as a folder which summarizes and documents every home visit made daily, the length of the visit, its location, the topics covered, the skills used by the CHW, and the perceived impact. The console reports also include the weight and length ratings of the child visited plotted on a growth chart. The supervisors do not change this information collected by the CHW on mobile phones, but merely review and use it for supervision and management of the CHWs. Supervision will facilitate CHW skill improvement over time. CHWs will be able to call mothers to check on illnesses such as diarrhea, provide reminders for immunizations and follow-up medical visits for HIV ARV or CD4 counts, as well as to make supportive calls to encourage depressed mothers to engage in pleasant events and a range of other motivating activities. Support, reminders, and monitoring will be the three primary functions that the supervisors will be encouraged to perform. Supervisors will not provide specific scripts or text messages for the CHWs. At each visit, the CHW enters a child’s weight and height in the mobile phone, which is translated into a WHO growth chart that both the mother and the CHW can review. The growth chart is brightly colored, clear, with a yellow color when the baby is in the desirable height-weight zone.

There will be a “flag system” to prompt the CHW and supervisor when visits are due by the CHW and when a follow-up is needed. In addition to gathering real-time data on the health of the household, the mobile phone monitoring system automatically lists all follow-up visits needed for each week. For example, the CHW is expected to reach all households in their catchment area within a maximum of a 6-month period. Pregnant mothers are to be visited monthly and every 2 weeks within the last 6 weeks of pregnancy. A household with a severely malnourished or stunted child must be visited weekly. A household with a child within one SD of the normal height and weight only needs to be visited monthly. MLHs must be visited more often both in pregnancy and post-birth, especially if there has been no reengagement in care following childbirth (See Table 2).

**Table 2:** Identification of responsibility for administrative and implementation functions with community health workers (CHWs)

	Standard Care	Accountable Care
Hiring	Local government	Local government
Salary	Local government	Local government
Training	Initial Philani Intervention Model (PIM) training	Initial PIM training and mobile phones provided
Visit documentation	Log sheet	CHW will log home visits in real time on mobile phones and enter information about the pregnant women in a folder for follow up purposes
Supervision	Local government	PIM
Type of supervision	Log sheet	Random supervision visits every 2 weeks including real-time and data-informed feedback; support and reminders will be sent to CHW and supervisors via mobile phones (“flag system”)

### Data analysis

A composite score will be calculated based on measures from birth to 24 months post-birth, similar to our composite measure in our previous RCT in Cape Town [33, 34]. It will be a sum of assessments of the attainment (1) or not (0) of well-being from 10 indicators of MCH. Outcomes will be assessed as present (1) or absent (0). Base rates influence the number of significant outcomes needed on individual domains to yield a significant result. To examine overall differences between MCH outcomes in the SC in contrast to the AC, a single index summing the 10 indicators will be calculated as the sum of 1 s, reflecting a score above the median (e.g., on months of breastfeeding) or present (getting all immunizations, attending > 3 antenatal visits), or 0 s, reflecting a score below the median or not present (e.g., no child grant, no maternal HIV testing in pregnancy).

The primary analyses will examine cumulative change across multiple indicators of maternal and infant health. For outcomes only assessed at 24 months, differences between AC and SC conditions will be examined using random effects regression models to control for neighborhood clustering. Differences between outcomes assessed at baseline, 6, 15, and 24 months will be explored using multi-level models adjusting for repeated measures and neighborhood effects. Data analysis will be conducted using SAS software version 9.2 [50]. Linear regression will be used for continuous variables, and logistic regression and Poisson/negative binomial regression will be implemented for binary and count outcomes, respectively. Logistic regression will be used for longitudinal binary outcomes.

#### Moderators

In addition to the overall impact of PIM on children and mothers, we will also examine the differential impact of PIM on subpopulations of children and mothers based on poverty and health-related characteristics at baseline (e.g., MLH, adolescent mothers, women using alcohol, depressed women); i.e., we will test the moderating effects of these baseline characteristics. Separate models will be run for each moderator. The number of visits, ratings of the CHW supervisor on competency, content of the visits, skills used, frequency of phone contacts logged in real-time on mobile phones and verified by such systems as GPS, and random observations every 2 weeks are also moderators and will be analyzed in a similar fashion as above.

#### Mediators

We will also use Structural Equation Models to examine three pathways, namely the extent to which the effect of PIM on child outcomes is mediated through maternal behavior (e.g., caretaking); the extent to which a direct (not mediated) effect of PIM on child outcomes is also present; and the direct effect of maternal behaviors on child outcomes.

#### Power calculations

The primary outcome is the single outcome measure which sums the number of significant individual outcome measures and calculates the number of tests necessary to indicate a significant difference. All individual outcomes are based on an alpha = 0.05. There is sufficient power of 0.8 to detect a small effect size of 0.21 overall omnibus test between the SC and AC by 24 months. An intraclass area correlation of 0.01 and 80% retention is assumed. For subgroup analyses whose sample size is 1/2 (1/3) of the full data set, there is power to identify effect sizes of 0.31 (0.38) for alpha = 0.05, power = 0.8, supporting the detection of medium-small or better effect sizes in subgroups of size 1/2 or 1/3 of the population. Longitudinal analyses of child data beyond two time points (i.e., more than 6 and 15 months) may add modest increases to the power. For analyses of mothers’ data, longitudinal analyses will be conducted over all pre- and post-natal time points and tests of level shift for these analyses should have greater power than for child analyses, which will tend to be over fewer time points.

### Publications and community involvement

Dissemination of findings will be provided to the community through meetings, which will serve as an opportunity for researchers to discuss results and receive feedback from community members. Publications will also be produced regularly and made publicly available in accordance with the National Institutes of Health’s Open Access Policy.

## Discussion

The proposed RCT will evaluate whether routinely implementing training as well as accountability structures to monitor CHW behavior and MCH outcomes with mobile phones and providing data-informed supervision will result in government services becoming efficacious. South Africa remains an important site to demonstrate scaling of efficacious home visits by CHWs, especially for MLH. It is critical to include all mothers in home visiting so that MLH will not be stigmatized and rejected. In addition, HIV commonly co-occurs with depression, alcohol abuse, intimate partner violence, and malnutrition in South Africa [10, 11, 15]. South Africa did not meet the MCH Millennium Development Goals 4 (child health), 5 (maternal health), or 6 (infectious diseases) [51, 52]. This project will be a strong test to assess if the government-employed CHWs can create efficacious outcomes, within a challenging work culture that currently does not have the resources to provide ongoing in-service training nor vigorous structures to maintain what is delivered during home visits.

The importance of identifying effective training models is highlighted by the recent Accra Call for Action for all governments to fund CHWs to expand their capacity to deliver evidence-based interventions (EBIs) [53]. Current scientific practice requires that an HIV prevention program be validated by two RCTs to document that it is an EBI; then, the manuals of specific HIV EBIs must be replicated with fidelity by providers or other researchers for a specific population and setting [54]. While the scientific community may require replication with fidelity, these current scientific norms are often not realistic [55], especially for LMICs. Rather than aiming for replication with fidelity to a manual, CHWs in the PIM are taught the foundational theory of behavior change and skills common to 80% of all child and adolescent mental health EBIs [56].

In South Africa, CHWs are taught to address and encourage health regarding alcohol, HIV, malnutrition, and depression. For each topic domain, a manual is written, framing the issue and providing some information to be applied in everyday life, building skills, establishing a social network and a set of implicit rewards to maintain the behaviors, and addressing the barriers to implementation. The manuals become prototypes that must be adapted and tailored by the CHW. If CHWs cannot become socially skilled problem solvers, no EBI is likely to be efficacious. The goal of training is to provide these skills and allow substantial tailoring, building on the common theory, principles, and skills shared across EBIs. Diffusing an EBI in this manner will be a disruptive innovation to standard practice in implementation science. By establishing training, monitoring, and supervision structures of the government’s CHWs, significantly improved MCH outcomes over 5 years are predicted. The results of this RCT will be applicable to the existing 1.2 million CHWs globally, to the 50% of Africa that is similarly deeply rural.

## Trial status

At the time of manuscript submission, recruitment had not begun.

## Additional file

Additional file 1: SPIRIT 2013 Checklist: Recommended items to address in a clinical trial protocol and related documents. (DOC 121 kb)

## Abbreviations

AC: accountable care condition
ARV: antiretroviral therapy
CHW: community health workers
EBI: evidence-based intervention
MCH: maternal and child health
MLH: mothers living with HIV
PIM: Philani Intervention Model
RCT: randomized controlled trial
SC: standard care condition
WHO: World Health Organization
